# Impact of left-behind duration on depression and anxiety in rural adolescents from mountainous areas and the moderating role of community resilience post-earthquake

**DOI:** 10.3389/fpsyt.2025.1606762

**Published:** 2025-07-07

**Authors:** Yong Xiang, Hao Yin, Jiaocan Yang, Donghu Chen, Jingjing Zhang

**Affiliations:** School of Architecture and Civil Engineering, Xihua University, Chengdu, China

**Keywords:** mountainous areas, earthquake impact, rural adolescents, depression, anxiety, community resilience

## Abstract

**Introduction:**

Adolescents in the mountainous regions of western Sichuan, China, are frequently exposed to earthquake disasters and often experience prolonged separation from parents due to labor migration. Previous studies have shown that both earthquake exposure and left-behind experiences negatively impact adolescent mental health, but the moderating role of community resilience remains underexplored.

**Methods:**

This study conducted a cross-sectional survey in 2023 among 541 rural adolescents (aged 12–18) affected by the 2022 Luding earthquake. Standardized instruments — the Kutcher Adolescent Depression Scale (KADS-6), the Generalized Anxiety Disorder Scale (GAD-7), and the 10-item Conjoint Community Resiliency Assessment Measure (CCRAM-10) — were used to measure depression, anxiety, and perceived community resilience, respectively. Moderation analysis was conducted using Hayes' PROCESS macro (Model 1), with simple slopes and Johnson-Neyman techniques applied.

**Results:**

The duration of left-behind status was significantly and positively associated with depressive and anxiety symptoms. Perceived community resilience was negatively associated with these outcomes and moderated the relationship between left-behind duration and mental health. Specifically, higher levels of community resilience attenuated—but did not eliminate—the adverse effects of prolonged parental absence.

**Discussion:**

Findings highlight the dual burden of disaster exposure and family separation on adolescent mental health in rural mountainous settings. Community resilience, encompassing leadership, preparedness, trust, and social cohesion, offers partial buffering effects. The results underscore the need for targeted psychological interventions and community-based resilience building to protect vulnerable youth in disaster-affected areas.

## 1 Introduction

Recent years have witnessed a pronounced increase in the global frequency of natural disasters and extreme meteorological events (1–3). From 2000 to 2019, the globe experienced 7,348 major disaster events, which collectively resulted in approximately 1.23 million fatalities, affected around 4.2 billion individuals, and incurred economic damages estimated at nearly $2.97 trillion. This period shows a significant escalation compared to the preceding two decades (1980-1999), during which there were 4,212 recorded events, about 1.19 million deaths, approximately 3.25 billion people impacted, and economic losses totaling $1.63 trillion (4). Earthquakes, characterized by their abrupt and catastrophic nature, not only threaten economic assets and human lives but also significantly impact mental well-being. The psychological repercussions of such events can be profound, potentially leading to long-term mental health disorders (5, 6). The primary reason for this is that earthquake survivors often experience intense psychological distress due to the life-threatening nature of the event, economic losses, and the grief of losing loved ones. This distress may lead to a range of health complications, encompassing both immediate and enduring physical and psychological conditions, including PTSD, depression, anxiety, insomnia, and profound grief (7–9). Additionally, earthquakes may force survivors to relocate urgently, exacerbating the inequalities faced by vulnerable groups (10, 11). Consequently, short-term mental health issues among some survivors may evolve into long-term psychological disorders (12).

In response to the damage caused by disasters, numerous scholars have conducted extensive research on various dimensions such as disaster exposure, emergency response capacity, urban disaster resilience, and social vulnerability to disasters, encompassing social, economic, and environmental aspects (13–16). These studies have proposed effective disaster prevention and mitigation measures, which are beneficial in reducing disaster losses and supporting sustainable societal development. However, most of these studies have primarily focused on the physical impacts on survivors, with relatively less attention given to the psychological aspects. Earlier studies have demonstrated that catastrophes profoundly affect survivors’ psychological well-being, often manifesting as depression and anxiety in the aftermath of such incidents (12, 17–19). As prevalent mental disorders, depression and anxiety affect a large portion of the global population (20). The World Health Organization reports that around 322 million individuals globally are affected by depression, and an additional 264 million experience anxiety disorders (21). China and India have particularly high numbers of depression cases (21). Following extreme events, women are more susceptible than men to developing anxiety and depression symptoms (22–24).

Earthquakes exert persistent and profound impacts on both the physical and psychological well-being of affected individuals (25, 26). Research conducted by Khachadourian et al. (27) pathological symptoms initiated by the quake severely disrupted the lives of these survivors. Moreover, numerous studies have demonstrated a direct relationship between the extent of earthquake exposure and the manifestation of depressive and anxiety symptoms. Additionally, several studies have indicated a positive correlation between the degree of earthquake exposure and the symptoms of depression and anxiety (28–30), while a negative correlation has been found between these symptoms and the support provided by the government, family, and friends (31). The study by Hu et al. (32) assessing survivors’ mental health 6 and 18 months post-2008 Wenchuan earthquake identified significant predictors of outcomes: loss of loved ones, property damage, and post-disaster psychosocial support. A literature review examining mental health implications post the 2011 Great East Japan Earthquake underscored the importance of incorporating disaster psychiatry within frameworks of emergency preparedness and response (33). However, the psychosocial issues induced by disasters extend beyond mental health disorders and include the stigmatization and discrimination faced by survivors (34, 35). For disaster victims, a harmonious and supportive community environment is crucial for the recovery from psychological disorders (36).

Community resilience fundamentally encapsulates a community’s inherent capacity to not only endure and mitigate the immediate impacts of adversities but also to effectively rebound and initiate recovery processes following such disruptions (37, 38). Plough et al. (39) suggest that community resilience encompasses both preparedness for emergencies and the establishment of a socially supportive environment. There is a close relationship between sustainable community development and residents’ mental health issues (40, 41). However, sustainability, as a broad concept, is inherently future-oriented, and the future is often characterized by significant uncertainty (42). Resilience, on the other hand, provides the ability to resist and recover from uncertain future threats and disruptions. Research has shown that community resilience contributes to sustainable community development (43) and is negatively correlated with psychological symptoms such as anxiety and depression (44). Therefore, high levels of community resilience can alleviate mental health issues among residents (45, 46). Specifically, community resilience reflects multiple key dimensions, including leadership, collective efficacy, emergency preparedness, place attachment, social trust, and social relationships, which together build the community’s comprehensive capacity to respond to disasters, mobilize resources, and promote the psychological well-being of its members (37).

The relationship between disaster exposure and mental health, as well as the profound impact of being left-behind on individual psychological well-being, has become a critical topic in psychology, sociology, and public health, garnering extensive research attention (6, 7). However, studies focusing on rural mountain residents are relatively scarce, particularly concerning adolescents in these areas. Adolescents left behind by parents who migrate for work often lack consistent parental companionship, making them more susceptible to mental health issues (47). The duration of being left-behind has been identified as an independent risk factor for adolescent mental health. During adolescence, being left- behind not only represents a challenge in adapting to changes in family structure but also has long-term and profound implications for physical and mental health in adulthood. This impact becomes more pronounced as the duration of being left-behind increases (48). Despite significant advancements in societal and economic development that have improved care for left-behind children in recent years, the challenges faced by left-behind adolescents in mountainous regions remain substantial. Research indicates that if adolescent psychological disorders are not promptly addressed, they may develop into clinical conditions (49).

Although existing studies have confirmed that individuals’ perceived community resilience plays a role in the relationship between psychological health and disaster exposure (45, 50), its moderating effect on the association between the duration of left-behind status and adolescent mental health has yet to be systematically explored. Most research has focused on the direct impact of disaster exposure on mental health, overlooking the potential moderating function of perceived community resilience as a critical psychosocial resource. More importantly, left-behind adolescents face unique psychological stress and adaptive challenges due to prolonged parental absence, resulting in pronounced vulnerability and long-term mental health issues. However, this population remains insufficiently addressed in the current literature. This research gap limits a deeper understanding of post-disaster mental health recovery mechanisms among left-behind adolescents and hinders the development of scientifically grounded and targeted intervention strategies. Therefore, it is imperative to investigate the moderating potential of perceived community resilience in mitigating the negative effects of left-behind status, in order to provide a more robust theoretical foundation and practical framework for post-disaster psychological interventions targeting adolescents.

This study conducted a survey among rural adolescents in severely affected areas of Luding County, Sichuan Province, collecting data on depression and anxiety symptoms, community resilience, and the duration of parental absence. Using Hayes’ (2013) PROCESS macro model (Model 1), the study analyzed the moderating role of community resilience in the relationship between parental absence duration and adolescent mental health. Simple slope analysis and the Johnson-Neyman technique were used to examine moderation effects, with corresponding plots generated to illustrate the findings.

## 2 Methods

### 2.1 Participants

In 2022, a 6.8 magnitude earthquake struck Luding, resulting in 117 fatalities and 3,275 injuries, with direct economic damages amounting to 15.48 billion RMB. One year after the earthquake, we conducted a cross-sectional survey in Moxi Town, Detuo Town, and Yanzigou Town, all within 20 kilometers of the epicenter in Luding County. The survey involved 617 rural adolescents who had experienced the earthquake and came from severely affected areas, ensuring a representative sample. Out of these, 541 completed questionnaires were analyzed achieving an 87.7% response rate, as detailed in **Table 1**. Moxi Town, Detuo Town, and Yanzigou Town are located in the remote rural mountainous areas of Luding County. Despite the rapid development of tourism in the mountainous areas of Sichuan in recent years, which has reduced the proportion of left-behind children, this issue remains significant in the region. Therefore, to investigate the impact of prolonged exposure to earthquake conditions on the mental health of rural adolescents, a substantial sample of youths was selected for the survey in these areas.

**Table 1:** Demographic characteristics of the study population.

Characteristics	Subgroup	N	%
Sex			
	Male	245	45.29
	Female	296	54.71
Age			
	12-18	541	100.00
Duration of stay for left-behind adolescents			
	None	188	34.74
	<6months	88	16.27
	<6months-1years	113	20.89
	<1years-1.5years	73	13.49
	<1.5years-2years	51	9.43
	+2years	28	5.18
Disaster exposure			
	No Harm and Loss	174	32.16
	Indirect Exposure	221	40.85
	Direct Non-fatal Injury	104	19.22
	Severe Direct Exposure	33	6.10
	Extreme Exposure	9	1.70

No Harm and Loss: The individual has not sustained any physical injuries, nor witnessed others being injured, and property remains undamaged.

Indirect Exposure: No direct injuries, but witnessed others trapped or partial property damage.

Direct Non-fatal Injury: The individual has sustained minor injuries or witnessed others receiving minor injuries.

Severe Direct Exposure: The individual was trapped or sustained moderate injuries, or witnessed bloody scenes.

Extreme Exposure: The individual has suffered severe injuries, lost loved ones, or witnessed deaths.

**Figure 1:**
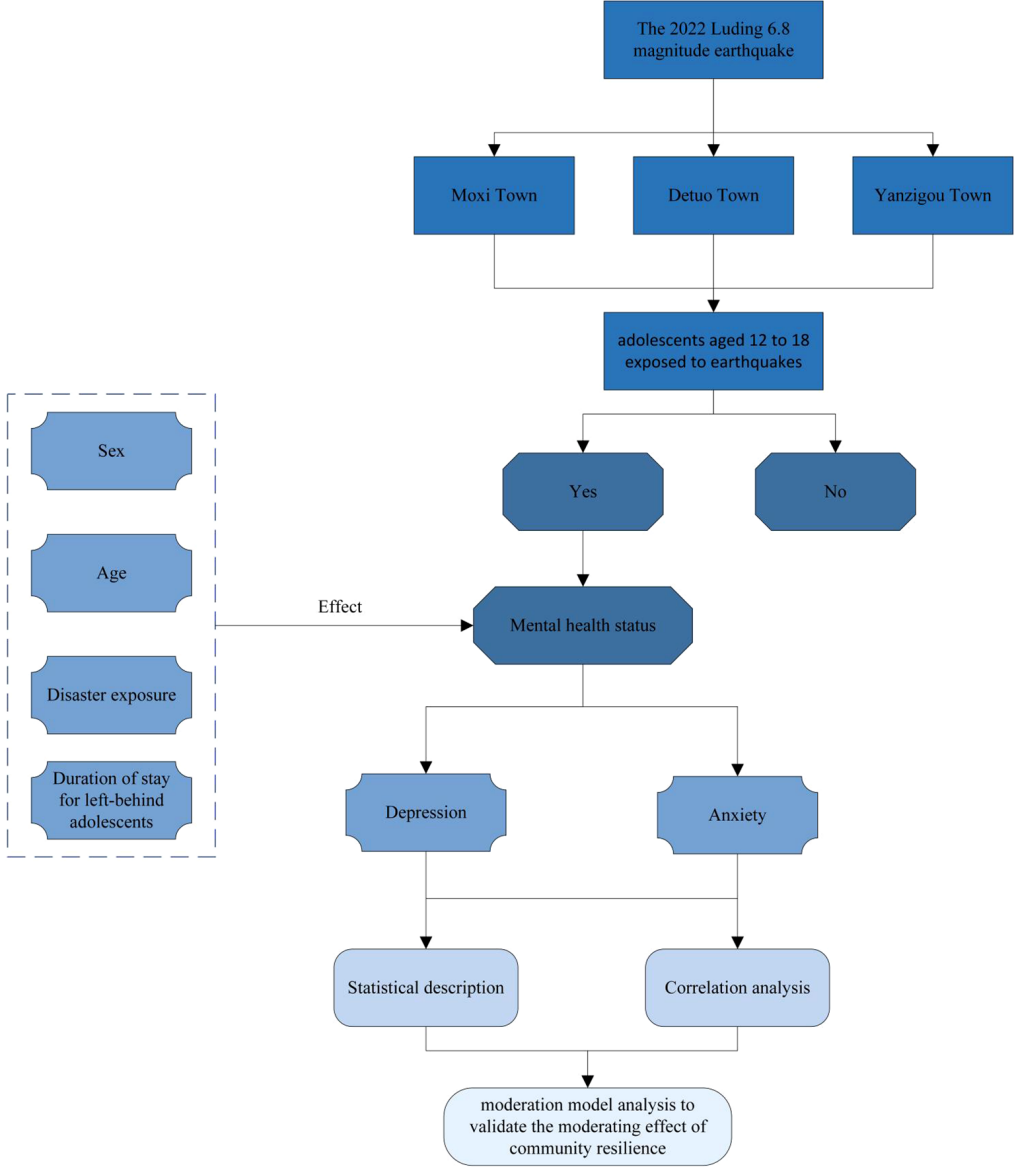
Framework for the study of adolescent mental health status post-earthquake exposure.

To enhance the scientific rigor and validity of this survey, we meticulously considered the unique circumstances of rural children in mountainous areas, as illustrated in the research design shown in **Figure 1**. Given that some children may have relocated to urban areas with their parents during their early childhood (51), only to return to attend middle or high school, or that some might have been too young during natural disasters such as earthquakes to have a clear recollection of the events, their assessment of community resilience could lack precision. Accordingly, our study design specifically excluded two key groups: children who did not directly experience the effects of the 2022 Luding earthquake, and children under the age of 12. This approach was aimed at focusing on adolescents who have firsthand experiences and can more accurately reflect the state of community resilience, thereby enhancing the precision of the research.

This study adheres to the ethical standards set forth in the Declaration of Helsinki and has received approval from the ethics committee. It permits the participation of middle school and high school students, who, along with their legal guardians, have provided informed consent.

### 2.2 Measures

The questionnaire consists of two sections. The initial section gathers essential data from participants, such as gender, age, the length of time left behind, and their exposure to earthquake disasters. The second part includes the Adolescent Depression Rating Scale (KADS-6) and the Generalized Anxiety Disorder Scale (GAD-7), as well as the Community Resilience Assessment Scale (CCRAM-10). These instruments are utilized to evaluate symptoms of depression, anxiety, and the community’s perceived resilience level.

#### 2.2.1 Depression

The severity of depressive symptoms is measured using the KADS-6, which employs a four-point rating scale (0-3) to assess the respondent’s depression symptom over the past week (52). The total score ranges from 0 to 18, with a cutoff point of 6. KADS-6 is designed for screening individuals who may have depressive symptoms, rather than serving as a formal diagnostic result. KADS-6 has been found to achieve 92% sensitivity and 71% specificity in measuring severe depression, demonstrating superiority relative to other diagnostic tools (53). KADS-6 is recognized as an effective and efficient tool for diagnosing Major Depressive Episodes (MDE) in adolescents (53). The scale’s Cronbach’s alpha is 0.83.

#### 2.2.2 Anxiety

This research employs the GAD-7 to evaluate anxiety symptoms in adolescents. The GAD-7 employs a four-point scale (0-3) to measure the impact of anxiety-related issues over the previous two weeks. The total score ranges from 0 to 21, with a cutoff value of 10 (54). This scale has been proven to have good reliability in measuring levels of anxiety symptoms (55) and is an effective tool for assessing anxiety symptoms in adolescents (56). The Cronbach’s alpha for this scale is 0.84.

#### 2.2.3 Community resilience

This study assesses the self-perceived level of community resilience using the Community Resilience Assessment Measure (CCRAM-10). This tool includes representative items such as *“There is mutual assistance and people care for one another”* and *“Residents in my community trust each other.”* The CCRAM-10 is commonly applied to investigate the connections between community resilience and several factors, including local government information dissemination (50), energy insecurity (57), disaster preparedness, emergency response (58), and perceived stress (59). Additionally, earlier studies have verified the suitability of the CCRAM-10 tool’s original version across different cultural contexts in China (60).

CCRAM-10 is a condensed version of the CCRAM-28, but it has been found to be more effective in measuring community resilience (37). The tool includes 10 questions, each assessed using a 5-point Likert scale (1-5), resulting in an overall score that varies from 10 to 50. A higher score indicates greater resilience within the community. The Cronbach’s alpha for this scale is 0.96.

### 2.3 Moderation model

This study employed IBM SPSS 27 for data analysis, using descriptive statistics to examine participant characteristics. Pearson correlation coefficients were calculated to explore associations among the primary variables. Covariates included disaster exposure, gender (male = 0, female = 1), and age (12–18), which represent key demographic factors. The independent variable was the duration of being left behind, while the dependent variables were depression and anxiety. Community resilience served as the moderating variable.

To test the moderating role of community resilience in the relationship between the duration of being left behind and mental health outcomes, Hayes’ (2013) PROCESS Macro Model 1 was utilized (61). The simple slopes analysis was conducted to evaluate the conditional effects of the independent variable at low, medium, and high levels of the moderator, with corresponding simple slopes plotted. While simple slopes analysis provides valuable insights, it may be less precise in the presence of skewed data or uneven distributions, potentially limiting its ability to capture moderating effects accurately. To address these limitations, the study also employed the Johnson-Neyman technique to assess the varying impact of the independent variable on the dependent variables across the entire range of the moderator. This approach identifies the specific values of the moderator at which the effects are statistically significant. The Johnson-Neyman technique offers a more nuanced and precise understanding of moderating effects, particularly for datasets characterized by skewness or uneven distributions, making it a robust complement to the simple slopes analysis.

In summary, this research explores the mechanisms by which community resilience moderates the prediction of adolescent mental health outcomes based on the duration of being left-behind following disaster exposure. It clarifies the mechanisms by which disaster exposure affects adolescent mental health and its individual differences. This contributes to targeted interventions and support for adolescents post-disaster, thereby enhancing their psychological resilience and overall well-being, offering empirical support and theoretical guidance to prevent or reduce post-disaster mental health issues among adolescents.

## 3 Results

**Table 2** presents the descriptive statistics and correlation analysis of the variables. The results reveal significant correlations among the key variables (p <.001). Specifically, depression and anxiety show a positive correlation with each other, and both are positively correlated with the duration of stay for left-behind adolescents and negatively correlated with perceived community resilience. Notably, a robust positive correlation exists between the duration of stay for left-behind adolescents and depression. Additionally, depressive and anxiety symptoms exhibit strong negative correlations with perceived community resilience. Conversely, the duration of stay for left-behind adolescents shows a negative correlation with perceived community resilience.

**Table 2:** Descriptive statistics and correlation analysis.

Variable	Mean (SD)	1	2	3	4
1.Depression	3.83 (2.29)	1			
2.Anxiety	3.74 (2.46)	0.669***	1		
3.Duration of Stay for Left-Behind Adolescents	1.62 (1.55)	0.515***	0.468***	1	
4.Community Resilience	37.97 (7.59)	-0.605***	-0.567***	-0.290***	1

***p <.001.

### 3.1 Community resilience as a moderator: linking the duration of adolescents being left-behind to depression

The model explained 54.34% of the variance in depressive symptoms (F = 90.607, p <.000), with the interaction effect accounting for an additional 0.66% of the variance (F = 7.736, p <.005). Adolescents in rural mountainous areas exhibited relatively high levels of depressive symptoms following earthquake exposure. The duration of left-behind status, age, and degree of disaster exposure were all positively correlated with the severity of depressive symptoms, with female adolescents being more susceptible than males. As the duration of being left behind increased, depressive symptoms became significantly more pronounced. Higher levels of community resilience were negatively associated with depressive symptoms. Based on the analysis results presented in **Table 3**, sex (β = 0.036, p < 0.01), age (β = 0.193, p < 0.001), disaster exposure (β = 0.066, p <.001), duration of left-behind status (β = 0.217, p <.001), community resilience (β = -0.101, p <.001), and their interaction (β = -0.003, p <.01) all showed significant effects, highlighting the critical protective role of community support in mitigating depressive symptoms among left-behind adolescents.

**Table 3:** Moderating effects of community resilience on depression and anxiety among left-behind adolescents exposed to earthquakes.

Variable	β	se	t	LLCI	ULCI
Depression (KADS-6 Model)					
Sex	0.036	0.014	2.680**	0.010	0.063
Age	0.193	0.039	4.988***	0.117	0.269
Disaster exposure	0.066	0.015	4.320***	0.036	0.097
Duration of Stay for Left-Behind Adolescents (A)	0.217	0.041	5.260***	0.136	0.297
Community Resilience (B)	-0.101	0.016	-6.296***	-0.132	-0.069
AB	-0.003	0.001	-2.781**	-0.005	-0.001
Anxiety (GAD-7 Model)					
Sex	0.029	0.015	1.630*	0.005	0.054
Age	0.272	0.043	6.339***	0.188	0.356
Disaster exposure	0.130	0.017	7.622***	0.097	0.164
Duration of Stay for Left-Behind Adolescents (A)	0.222	0.046	4.869***	0.133	0.312
Community Resilience (B)	-0.085	0.018	-4.824***	-0.120	-0.051
AB	-0.004	0.001	-2.810**	-0.006	-0.001

Se, standard error, *p <.05, **p <.01, ***p <.001.

**Figure 2:**
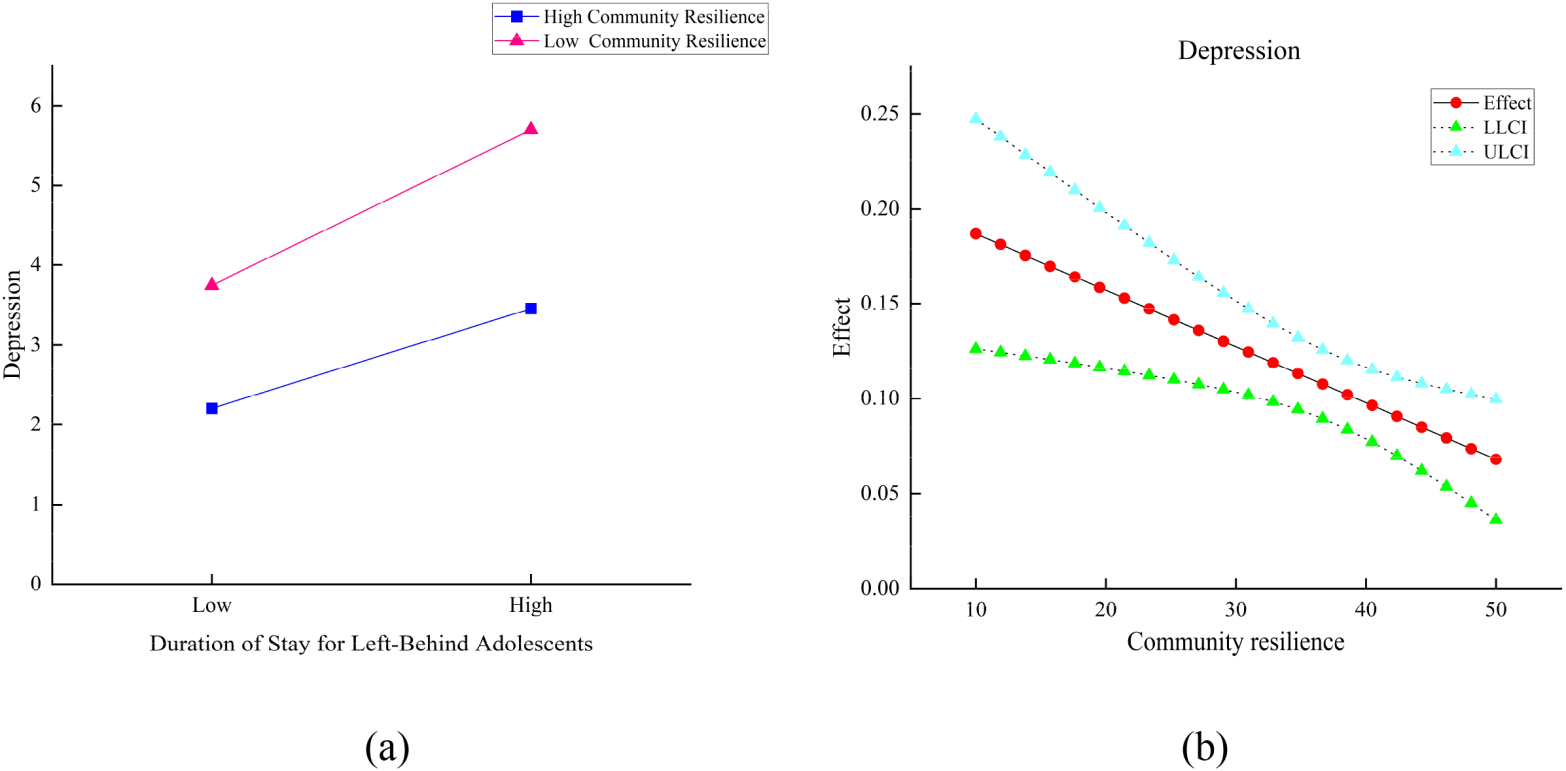
Simple slopes diagram **(a)** and Johnson-Neyman plots **(b)**: Community Resilience Moderating the Impact of Duration of Adolescents Being Left Behind on Depression. LLCI, Lower Level Confidence Interval; ULCI, Upper Level Confidence Interval. Effect: The effect of the duration of being left behind on depression at different levels of community resilience.

Furthermore, as indicated by the results in **Table 4**, across different levels of community resilience (low, medium, high), the influence of the duration of parental absence on depressive symptoms shows a gradually weakening trend with the increase in community resilience. Through the analysis of the simple slope plot and Johnson-Neyman plot in **Figure 2**, it was observed that the conditional effects of community resilience are significant when the scores range from 10 to 50. Specifically, in communities with lower levels of resilience, the duration of adolescent left-behind status significantly predicted an increase in depressive symptoms. This finding suggests that prolonged familial separation in less resilient communities exacerbates psychological distress. Conversely, in communities with higher levels of resilience, while the duration of left-behind status still positively predicts depressive symptoms, the strength of this effect is significantly attenuated. This indicates that stronger community resilience may partially buffer the negative psychological impacts of prolonged separation; even when the duration of left-behind status is extended, higher community resilience can mitigate the severity of depressive symptoms. This finding underscores the critical role of community resilience in moderating the adverse psychological effects of left-behind status on adolescent mental health.

**Table 4:** Slopes of the predictive relationships between duration of left-behind adolescents and depression, anxiety at different levels of community resilience.

Symptoms	Community Resilience	Conditional effect	se	t	LLCI	ULCI
Depression	Low	0.126	0.012	10.544***	0.103	0.150
	Middle	0.104	0.009	11.263***	0.086	0.122
	High	0.081	0.013	6.460***	0.057	0.106
Anxiety	Low	0.121	0.013	9.123***	0.095	0.147
	Middle	0.096	0.010	9.389***	0.076	0.116
	High	0.071	0.014	5.069***	0.043	0.098

Se, standard error, ***p <.001.

### 3.2 Community resilience as a moderator: linking the duration of adolescents being left-behind to anxiety

The model accounted for 51.44% of the variance in anxiety symptoms (F = 80.645, p <.001), with the interaction effect leading to a 0.72% change in R² (F = 7.894, p = .005). According to the results shown in **Table 3**, age (β = 0.272, p <.001), disaster exposure (β = 0.130, p <.001), and the duration of parental absence (β = 0.222, p <.001) were all significant positive predictors of adolescents’ anxiety symptoms, while community resilience (β = -0.085, p <.001) was significantly negatively associated with anxiety symptoms, indicating a buffering effect. Moreover, the interaction term between duration of parental absence and community resilience (β = -0.004, p <.005) demonstrated that community resilience significantly attenuates the negative impact of prolonged parental absence on anxiety symptoms, highlighting its protective role in mitigating psychological risks associated with family separation.

**Figure 3:**
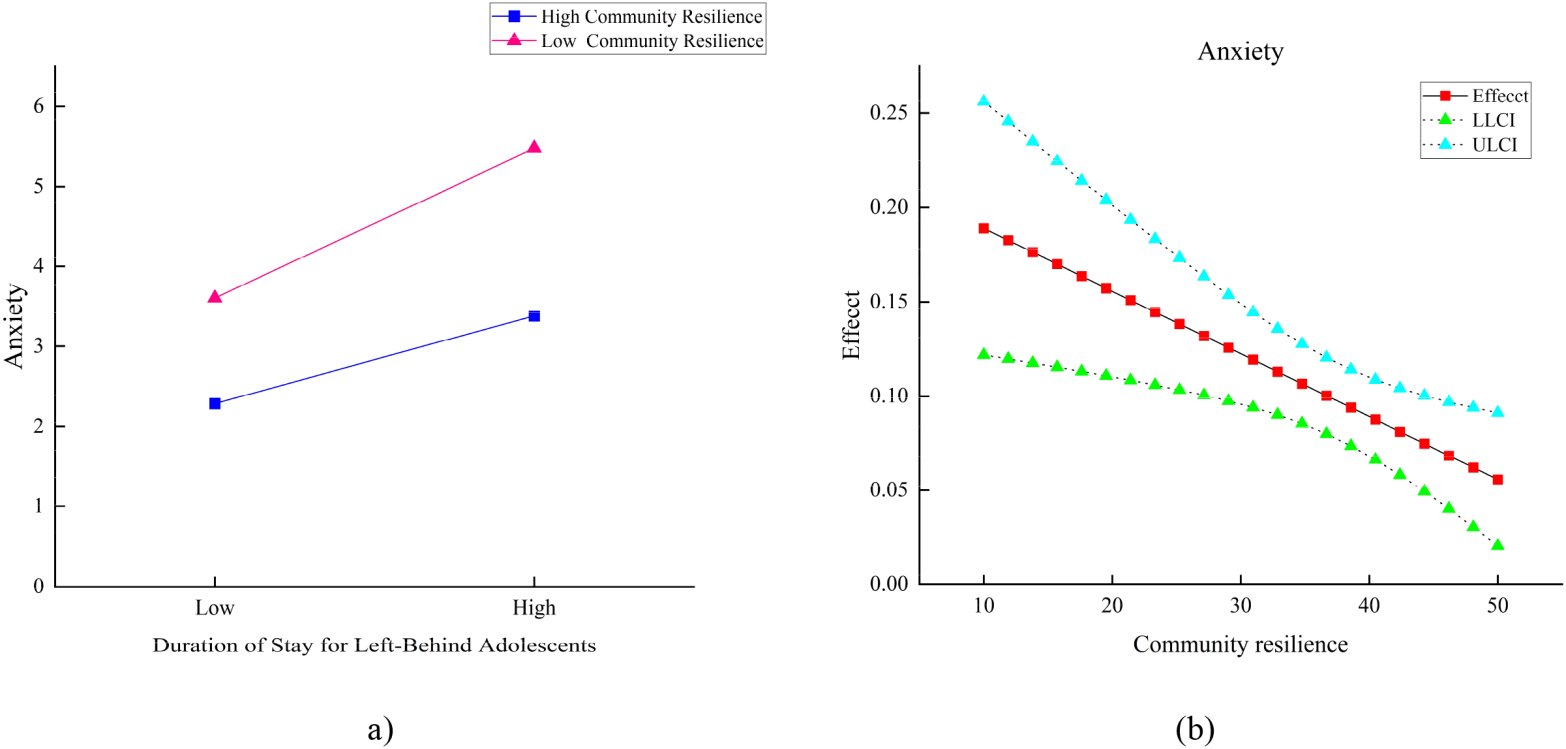
Simple slopes diagram **(a)** and Johnson-Neyman plots **(b)**: Community Resilience Moderating the Impact of Duration of Adolescents Being Left Behind on Anxiety. LLCI, Lower Level Confidence Interval; ULCI, Upper Level Confidence Interval. Effect: The effect of the duration of being left behind on anxiety at different levels of community resilience.

Furthermore, **Table 4** further demonstrates that across different levels of community resilience (low, medium, high), the impact of the duration of parental absence on anxiety symptoms gradually weakens as community resilience increases. The analysis of the simple slope plot and Johnson-Neyman plot in **Figure 3** revealed that the conditional effects of community resilience are significant when the resilience scores range from 10 to 50. In communities with lower levels of resilience, the duration of adolescent left-behind status significantly predicted an increase in anxiety symptoms. This suggests that prolonged familial separation in less resilient communities markedly intensifies anxiety among adolescents. Conversely, in communities with higher levels of resilience, although the duration of left-behind status continues to positively predict anxiety symptoms, the strength of this effect is significantly diminished. This finding indicates that higher community resilience may serve a protective role, mitigating the impact of prolonged separation on adolescent anxiety symptoms. It also suggests that community resilience can act as a buffer against the psychological effects of familial separation on adolescent anxiety.

In conclusion, the duration of parental absence among disaster-affected adolescents significantly predicts both depression and anxiety, and the model demonstrates good predictive capability. The findings indicate that the moderating effect of community resilience becomes more pronounced when resilience scores are 38 or higher. As the level of community resilience increases, the predictive impact of the duration of parental absence on depressive and anxiety symptoms gradually decreases. Moreover, although community resilience significantly moderates the relationship between the duration of parental absence and both depressive and anxiety symptoms at all levels, the impact of the duration of absence on these psychological symptoms remains significant. This suggests that the negative effects of prolonged parental absence on adolescent mental health cannot be entirely offset by community resilience.

## 4 Discussion

Earthquake disasters pose significant and urgent threats not only to human life, livelihood, and health but also have profound potential impacts on mental well-being. This study investigated the prevalence of depression and anxiety symptoms among rural adolescents in the Sichuan mountainous areas following exposure to earthquakes. Many of these adolescents are left behind for extended periods, often exceeding six months, as their parents seek employment opportunities in urban areas. Exposed to the aftermath of such disasters, many of these youths face both physical and/or psychological trauma, exacerbated by the lack of parental support and comfort (51). Based on this, the study identified the influence of the duration of being left behind post-disaster on depression and anxiety, and verified the moderating role of community resilience.

This study confirms previous findings that age and disaster exposure significantly impact symptoms of depression and anxiety (23, 26, 27). Furthermore, the influence of depression and anxiety symptoms among adolescents in mountainous rural areas varies by gender, with females being more susceptible to depression compared to males, consistent with earlier research (23, 24). The primary reason for this disparity lies in the traditional cultural practices of remote mountainous areas, where girls receive relatively less attention and support compared to boys. When faced with difficulties in life or emotional challenges, girls may feel more lost and helpless if their parents cannot provide timely support, making them more prone to negative emotions. Over time, this can lead to increased symptoms of anxiety and depression. Therefore, in remote rural areas, the role of socio-cultural context and family support in adolescent mental health cannot be overlooked, especially the lack of attention and support for female adolescents, which may be a crucial factor in their mental health issues.

In rural mountainous areas, parental lack of attention to children is influenced not only by gender differences but also by economic necessities. Often, parents are compelled to seek employment in urban areas to improve their family’s living standards, leaving them unable to be physically present with their children. Consequently, in the aftermath of disasters, the absence of parental support renders children more susceptible to emotional trauma. Previous studies have primarily focused on the impact of disaster exposure on the mental health of adolescents who are left behind or not, overlooking how mental health conditions may vary with the duration of being left behind (51, 62). Building on this, our study reveals a positive correlation between the duration of stay for left-behind adolescents and symptoms of depression and anxiety. This finding aligns with the results from Wang et al. (62), who studied different groups of adolescents—including those left behind, migrant, and local—finding that left-behind adolescents are more vulnerable to adverse mental health outcomes. Moreover, our research also confirms that the longer the duration of absence due to parental migration in the context of earthquake disasters, the higher the likelihood of poor mental health outcomes, suggesting a more pronounced negative impact on mental health with increasing duration of absence.

The study results show a negative correlation between the duration of stay for left-behind adolescents and community resilience. This reflects that the stronger the resilience perceived in their communities, the more it mitigates the impacts of prolonged absence on adolescents, indicating that community resilience can buffer the effects of extended durations of parental absence (45, 46). Community resilience encompasses effective leadership, collective efficacy, preparedness to face risks and challenges, a profound attachment to place, and a high level of trust among community members (50, 58, 59). High community resilience implies that governmental organizations have enhanced response capabilities and can depend on a robust social infrastructure. In the event of disasters, such communities are capable of mobilizing social resources quickly and efficiently to support individuals or groups, and provide effective assistance through close collaboration between organizations and community members. The study finds that community resilience moderates the relationship between the duration of stay for left-behind adolescents and symptoms of depression and anxiety, effectively alleviating the impact of these mental health symptoms following exposure to earthquake disasters as the duration of absence increases.

Community resilience plays a crucial moderating role in the duration of youth left unattended and the manifestation of depression and anxiety symptoms following disaster exposure. Adolescents witnessing the severe destruction of homes and infrastructure during an earthquake often experience profound feelings of loneliness and helplessness, which are primary triggers for depressive and anxiety symptoms (7, 9). The risk of depression and anxiety significantly increases if adolescents remain without parental presence post-disaster (47). However, communities with high resilience can mitigate these adverse effects at multiple levels. Firstly, efficient disaster response capabilities allow for swift action post-disaster, not only hastening the restoration of basic order but also providing essential medical and psychological support to aid affected youths in timely treatment, thereby alleviating impacts on their mental health. Secondly, collective collaboration and cooperation enhance the social support networks within the community, enabling youths to feel cared for by neighbors and peers. This support helps them share experiences and express emotions post-disaster, thus alleviating feelings of isolation. Lastly, communities well-prepared for emergency situations ensure timely protection and psychological support for youths during disasters, reducing their panic and distress.

Furthermore, communities with high resilience often provide ongoing mental health counseling and educational services, assisting adolescents in better understanding and managing the emotional changes they experience post-disaster, and in recognizing symptoms of depression and anxiety (63). This comprehensive social support not only helps mitigate the negative impacts of disasters and parental absence on adolescent mental health but also teaches them how to cope with and manage post-traumatic emotional responses, reducing the risk of long-term psychological issues and enhancing their psychological resilience (64). Through these multi-level interventions, resilient communities effectively alleviate the psychological burdens faced by adolescents due to disaster exposure and prolonged absence, thereby decreasing the incidence of depressive and anxiety symptoms.

Although community resilience significantly moderates the relationship between the duration of left-behind status and symptoms of depression and anxiety, its moderating effect does not completely eliminate the association. Specifically, community resilience can somewhat weaken the impact of prolonged left-behind duration on depression and anxiety symptoms, but it is insufficient to fully offset this impact. The effect of prolonged left-behind duration on these symptoms remains significant, particularly after disaster exposure. The findings from the CCRAM-10 provide an effective framework for identifying communities with low resilience. This framework allows for the identification and prioritization of such communities, enabling targeted interventions. For example, communities with weak emergency preparedness can improve their emergency response capabilities through disaster preparedness drills, while communities with low social trust may require a range of social support measures to strengthen community networks and accumulate social capital. To effectively alleviate the psychological health issues of left-behind children in mountainous areas, a comprehensive school-based psychological health service system should be established based on specific local conditions. This system should include the following components: (1) Regular psychological health screenings, using standardized questionnaires, teacher observations, and individual interviews to promptly identify students experiencing psychological distress and provide targeted interventions; (2) Provision of regular psychological counseling services, with professional counselors offering personalized guidance to help students cope with stress, anxiety, and emotional difficulties; (3) Regular psychological health training for teachers, aimed at enhancing their ability to identify and intervene in psychological issues, particularly those related to the emotional needs and psychological characteristics of left-behind children, ensuring that teachers can effectively detect and address students’ psychological problems in their daily teaching.

**Figure 4:**
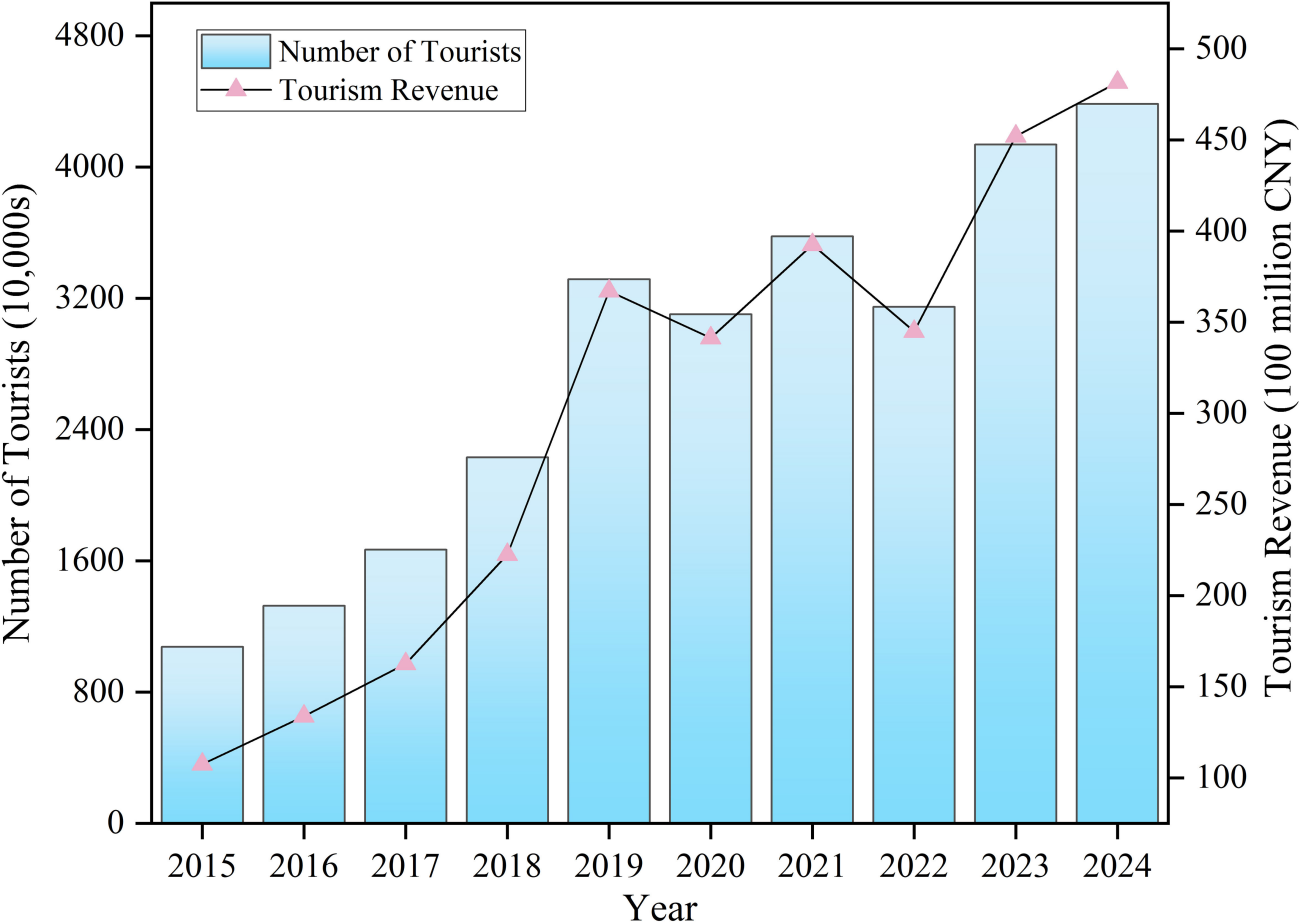
Tourism development trends in Ganzi Prefecture.

In addition, it is essential to leverage the unique natural landscapes and cultural resources of mountainous regions, while continuously strengthening policy support to promote rural tourism development. This can create more local employment opportunities, attract migrant workers to return home for entrepreneurship or nearby employment, and fundamentally reduce family separation, thereby alleviating the issue of left-behind adolescents. As shown in **Figure 4**, in recent years, the tourism industry in Ganzi Prefecture, where the study area is located, has exhibited a steady growth trend in both tourist arrivals and tourism revenue. Although there was a temporary decline in 2020 due to the impact of the COVID-19 pandemic, the industry has demonstrated strong resilience and recovery capabilities, becoming a key driver of regional economic development and family reunification. Today, tourism has become a pillar industry in Ganzi Prefecture, providing abundant employment opportunities for local residents across various sectors, including tour guiding, hotel services, catering, and transportation. This not only promotes economic growth but also plays a vital role in improving family structures and addressing the left-behind phenomenon.

## 5 Limitations

There are certain limitations inherent in the methodology of this study. This study focused on adolescents aged 12 to 18 who had experienced an earthquake and demonstrated sufficient cognitive capacity. While this ensured sample consistency, the narrow age range limits the generalizability of the findings. Accurate data on family income, an important factor in post-disaster mental health, was difficult to obtain in remote rural areas, restricting socioeconomic analysis. Key family structure variables, such as household size, caregiver type, and the duration of left-behind status, were not systematically differentiated, suggesting directions for future research. Additionally, secondary disasters common in mountainous areas, such as landslides and mudflows, were not considered, despite their potential to intensify psychological distress. As a cross-sectional study, this research could not capture the long-term mental health impacts or the evolving role of community resilience, highlighting the need for longitudinal designs in future work.

## 6 Conclusions

This study, which surveyed 541 rural adolescents in the mountainous areas of Sichuan, found that symptoms of depression and anxiety among left-behind adolescents significantly worsened with longer periods of parental absence. Additionally, the study identified that community resilience plays a crucial moderating role between the duration of being left behind and the severity of these psychological symptoms. Specifically, adolescents in highly resilient communities exhibited notably lower levels of depression and anxiety even when facing prolonged parental absence, in comparison to those in communities with lower resilience. However, community resilience alone does not entirely neutralize the adverse effects of being left behind on adolescent mental health. Consequently, it is advised that local governments implement post-disaster mental health interventions, focusing particularly on the psychological well-being of rural adolescents who experience extended periods of being left behind. This should include enhancing social support and care for these adolescents, as well as encouraging the return of rural residents through policies that support entrepreneurship or employment opportunities, thereby addressing the root causes of the left-behind phenomenon among adolescents in mountainous areas.

## Data Availability

The datasets presented in this article are not readily available because this study strictly adheres to ethical guidelines to ensure the privacy and confidentiality of the data of vulnerable rural adolescents. To ensure security and prevent information leakage, all data is encrypted and anonymously processed, and can only be accessed by the research team of this study. Requests to access the datasets should be directed to Hao Yin, yinhao126@stu.xhu.edu.cn.
